# Cellular source of hypothalamic macrophage accumulation in diet-induced obesity

**DOI:** 10.1186/s12974-019-1607-0

**Published:** 2019-11-14

**Authors:** Chan Hee Lee, Sung Hoon Shin, Gil Myoung Kang, Seongjun Kim, Jiye Kim, Rina Yu, Min-Seon Kim

**Affiliations:** 1 Asan Institute for Life Sciences, University of Ulsan College of Medicine, Seoul, 05505 Republic of Korea; 2 Department of Biomedical Science, Asan Medical Institute of Convergence Science and Technology, Asan Medical Center and University of Ulsan College of Medicine, Seoul, 05505 Republic of Korea; 3 Department of Food Science and Nutrition, University of Ulsan, Ulsan, 44610 Republic of Korea; 4 Division of Endocrinology and Metabolism, Department of Internal Medicine, Asan Medical Center and University of Ulsan College of Medicine, 88 Olympic-ro 43-gil, Songpa-gu, Seoul, 05505 Republic of Korea

**Keywords:** Obesity, Hypothalamus, Inflammation, Macrophages, Development

## Abstract

**Background:**

Obese mice on a high-fat diet (HFD) display signs of inflammation in the hypothalamic arcuate nucleus (ARC), a critical area for controlling systemic energy metabolism. This has been suggested as a key mechanism of obesity-associated hypothalamic dysfunction. We reported earlier that bone marrow-derived macrophages accumulate in the ARC to sustain hypothalamic inflammation upon chronic exposure to an HFD. However, the mechanism underlying hypothalamic macrophage accumulation has remained unclear.

**Methods:**

We investigated whether circulating monocytes or myeloid precursors contribute to hypothalamic macrophage expansion during chronic HFD feeding. To trace circulating myeloid cells, we generated mice that express green fluorescent protein (GFP) in their lysozyme M-expressing myeloid cells (LysM^GFP^ mice). We conducted parabiosis and bone marrow transplantation experiments using these animals. Mice received an HFD for 12 or 30 weeks and were then sacrificed to analyze LysM^GFP^ cells in the hypothalamus. Hypothalamic vascular permeability in the HFD-fed obese mice was also tested by examining the extravascular leakage of Evans blue and fluorescence-labeled albumin. The timing of LysM^GFP^ cell entry to the hypothalamus during development was also evaluated.

**Results:**

Our parabiosis and bone marrow transplantation experiments revealed a significant infiltration of circulating LysM^GFP^ cells into the liver, skeletal muscle, choroid plexus, and leptomeninges but not in the hypothalamic ARC during chronic HFD feeding, despite increased hypothalamic vascular permeability. These results suggested that the recruitment of circulating monocytes is not a major mechanism for maintaining and expanding the hypothalamic macrophage population in diet-induced obesity. We demonstrated instead that LysM^GFP^ cells infiltrate the hypothalamus during its development. LysM^GFP^ cells appeared in the hypothalamic area from the late embryonic period. This cellular pool suddenly increased immediately after birth, peaked at the postnatal second week, and adopted an adult pattern of distribution after weaning.

**Conclusions:**

Bone marrow-derived macrophages mostly populate the hypothalamus in early postnatal life and may maintain their pool without significant recruitment of circulating monocytes throughout life, even under conditions of chronic HFD feeding.

## Background

Chronic low-grade inflammation is commonly found in the adipose tissue, liver, skeletal muscle, pancreatic islets, and blood vessels of obese humans and animals [1, 2]. Mounting evidence has suggested that obesity-associated inflammation, so-called meta-inflammation, triggers glucose intolerance and type 2 diabetes by interrupting insulin signaling in insulin-target tissues [3, 4]. Macrophages are tissue-resident phagocytic cells and are thought to be a pivotal player in obesity-related inflammation in adipose tissues [5]. Macrophages that reside in adult healthy tissues are either derived from circulating monocytes or established before birth and then maintained throughout life independently of monocytes [6]. By contrast, most of the macrophages that accumulate at inflamed sites are typically derived from circulating monocytes [7]. Upon entering damaged tissue, monocytes undergo a series of changes to become macrophages. Monocytes are attracted to a damaged site through a chemotaxis response that is triggered by a range of stimuli including damaged cells, pathogens, and cytokines released by macrophages already at that site [6, 7].

The hypothalamus is a vital organ within the central nervous system (CNS) that governs systemic energy and glucose metabolism [8, 9]. Neurons in the hypothalamic arcuate nucleus (ARC), a small area near the median eminence (ME) lacking the blood-brain barrier (BBB) [10], sense periphery-derived metabolic signals such as leptin and insulin to maintain metabolic homeostasis [8, 9]. Similar to peripheral tissues, the obesity-related inflammation specifically occurs in the ARC and it has been suggested as an important mechanism of hypothalamic dysfunction during obesity progression [11, 12]. Microglia, which are cells that act like peripheral tissue macrophages, are thought to be the primary immune cells involved in this hypothalamic inflammation [11–13]. In particular, ARC microglia are readily activated in response to short-term exposure to an HFD and initiate a hypothalamic inflammatory response to saturated fatty acids [12–15]. Whereas adipose tissue macrophages are derived from monocytes of bone marrow origin [5], CNS microglia mostly arise from primitive hematopoietic cells in the yolk sac [16]. They migrate to the neuroepithelium during the early embryonic period and maintain their population through lifelong self-renewal.

Bone marrow-derived macrophages are also present in the hypothalamus [17, 18]. In contrast to the widespread distribution of microglia, we have recently reported that hypothalamic macrophages have a restricted distribution that includes the perivascular area, the leptomeninges, and the circumventricular organ ME [14]. We have also found profound macrophage activation and accumulation in the ARC of HFD-fed mice and reported a critical role of bone marrow-derived macrophages in HFD-induced hypothalamic inflammation [14]. In that study, we observed proliferating macrophages in the ARC and meningeal lining of obese mice [14]. However, it remained unclear whether these proliferating macrophages were derived from the systemic circulation or arose through the self-renewal of resident macrophages. In our present study therefore, we investigated the cellular source of hypothalamic macrophage expansion observed in a diet-induced obesity (DIO) mouse model.

## Methods

### Animals

C57BL/6 (C57) male mice were purchased from Orient Bio (Seongnam, Korea). Lysozyme M (LysM)-cre mice were cross-mated with mice harboring an enhanced green fluorescent protein (GFP) reporter allele with an upstream loxP-flanked STOP cassette (both from Jackson Laboratory, Bar Harbor, MA) to generate mice with myeloid cell-specific GFP expression (LysM^GFP^ mice). The animals were fed either a standard chow diet (CD; Samyang, Seoul, Korea) or a high-fat diet (HFD; 58% fat, Research Diet Inc., New Brunswick, NJ) and maintained under a controlled temperature (22 ± 1 °C) and a 12-h light-dark cycle (lights on 8 AM) with free access to food and water.

### Immunostaining

Mice were anesthetized with 40 mg/kg Zoletil® (zolazepam and tiletamine) and 5 mg/kg Rompun® and then perfused with 50 ml saline followed by 50 ml 4% paraformaldehyde (PFA) via the left ventricle. Whole brains were collected, fixed with 4% PFA for 24 h, and dehydrated in 30% sucrose solution until the brains sank to the bottom of the container. Coronal brain sections (20 μm thick) including the hypothalamus were then obtained using a cryostat (Leica, Wetzlar, Germany). Sections were stored at − 70 °C until immunostaining. Hypothalamic slices were permeabilized in 0.5% phosphate-buffered saline with Tween 20 for 5 min and blocked with 5% normal donkey serum at room temperature (RT) for 1 h and then incubated with primary antibodies against GFP (Aves Labs, #GFP-1010, 1:1000), the microglia marker Iba1 (Abcam, #Ab5076, 1:500), or vascular marker PECAM1 (BD Pharmingen, #550274, 1:200) at 4 °C for 16 h and then at RT for 1 h. After washing, slides were incubated with the appropriate Alexa-Flour 488- or 633-conjugated secondary antibodies (Invitrogen, 1:1000) at RT for 1 h. To stain the fenestrated vasculature, fresh-frozen hypothalamic slices were fixed in 4% PFA for 30 min, blocked with 5% normal donkey serum, and then incubated with anti-MECA32 antibody (BD Pharmingen, #550563, 1:200). The incubation protocol for primary and secondary antibodies was performed as described above. For nuclear staining, slides were treated with 4′,6-diamidino-2-phenylindole (DAPI, Sigma, #D9564, 1:10,000) for 10 min before mounting. Immunofluorescence was detected using confocal microscopy (Carl Zeiss 780, Germany). Quantitation of fluorescence intensity of Evans blue and MECA32 was performed throughout the entire rostro-caudal axis of the ARC using ImageJ.

### Vascular permeability test

To test hypothalamic vascular permeability, 3% Evans blue diluted in saline (Sigma, #E2129, 10 ml/kg) was administered via a tail vein to mice fed with a CD or an HFD for the indicated periods (*n* = 10). Whole brains were collected at 20 min post-injection and frozen with pre-chilled isopentane. Hypothalamic slices (20 μm thick) were sectioned using a cryostat, washed three times with phosphate-buffered saline, and incubated with DAPI prior to mounting. Evans blue fluorescence (excitation at 620 nm, emission at 680 nm) was imaged using a confocal microscope. Alternatively, the mediobasal hypothalamus (MBH) was harvested 20 min after the intravenous injection of Evans blue, weighed, and incubated in 300 μl formamide (AMRESCO, #0606-500 ml) at 70 °C for 24 h. Samples were centrifuged at 16,000×*g* for 45 min, and supernatants were collected to measure the optical density at 630 nm using a spectrophotometer (Eppendorf, Germany). Optical density readings were normalized to tissue weight. Hypothalamic vascular permeability was also tested using Alexa-Fluoro 680-conjugated albumin (Molecular Probes, #A34787, 10 mg/kg diluted in saline) (*n* = 3). Fluorescent-labeled albumin was administered via a tail vein. Whole brains were collected 20 min after injection, immediately frozen, sliced, and immunostained for PECAM1 as described above to confirm the extravasation of fluorescence-conjugated albumin. Immunofluorescence images were captured using a confocal microscope. All vascular permeability tests were performed during the early light period under free-feeding conditions.

### Parabiosis

Parabiont pairs of 13-week-old, weight-matched LysM^GFP^ mice and C57 male mice were surgically conjoined (6 pairs). Under anesthesia with Zoletil® and Rompun®, body skin from the lateral side of the parabionts was opened from the elbow and knee and sutured with a parabiont pair. Parabiosis success was confirmed by detecting the presence of LysM^GFP^ monocytes in the peripheral blood of C57 mice using flow cytometry (BD FACS Canto™). These blood samples were collected from the orbital vein of parabionts at 1 week post-surgery. At 6 weeks after parabiosis, animals were separated and perfused with 4% PFA. Brains were collected to observe LysM^GFP^ cells in the hypothalamus and choroid plexus of parabionts using GFP immunohistochemistry. Mice were fed either a CD or an HFD for 12 weeks (6 weeks before surgery and 6 weeks after surgery) before being sacrificed.

### Bone marrow transplant (BMT)

To generate bone marrow chimeric mice, 7-week-old C57 male mice were irradiated (6.5 Gy twice with 4-h intervals) with a head shield under anesthesia (*n* = 5). Twenty-four hours after irradiation, they were injected via the tail vein with bone marrow cells (3 × 10^6^ cells) harvested from the femur of LysM^GFP^ mice. At 6 weeks post-transplantation, we confirmed successful transplantation by detecting the presence of LysM^GFP^ monocytes in the peripheral blood using flow cytometry. Animals showing successful BMT were fed with a CD or an HFD for 12 or 30 weeks before being sacrificed. Brain, liver, and leg skeletal muscle (quadriceps femoris) were collected for GFP and DAPI immunostaining after cardiac perfusion with 4% PFA.

### Study of hypothalamic macrophages during development

Whole brains of embryos and neonates born to CD-fed LysM^GFP^ mothers were collected at the indicated ages (*n* = 2~5, from 2~3 different litters each time point). Mice younger than 7 days old were sacrificed without cardiac perfusion. Brains were fixed in 4% PFA for 24 h, dehydrated, sliced, and then subjected to GFP and Iba1 immunostaining as described above.

### Statistical analysis

Data are presented as means ± standard error of the mean (SEM). Statistical analyses were performed using SPSS version 23 (IBM Analytics, North Castle, NY). Statistical significance among the groups was tested using one-way or repeated measures analysis of variance (ANOVA) followed by a post hoc least significant difference (LSD) test, when appropriate. Significance was defined as *p* < 0.05.

## Results

### Increased vascular permeability in the ARC of HFD-fed mice

We hypothesized that hypothalamic macrophage accumulation in DIO mice may result from the enhanced recruitment of circulating monocytes. Most brain regions including the hypothalamus are immune-privileged as they are separated from the blood circulation by the blood-brain barrier (BBB) [19]. Thus, BBB disruption may be a prerequisite for the recruitment of circulating immune cells to the sites of neuroinflammation.

**Fig. 1:**
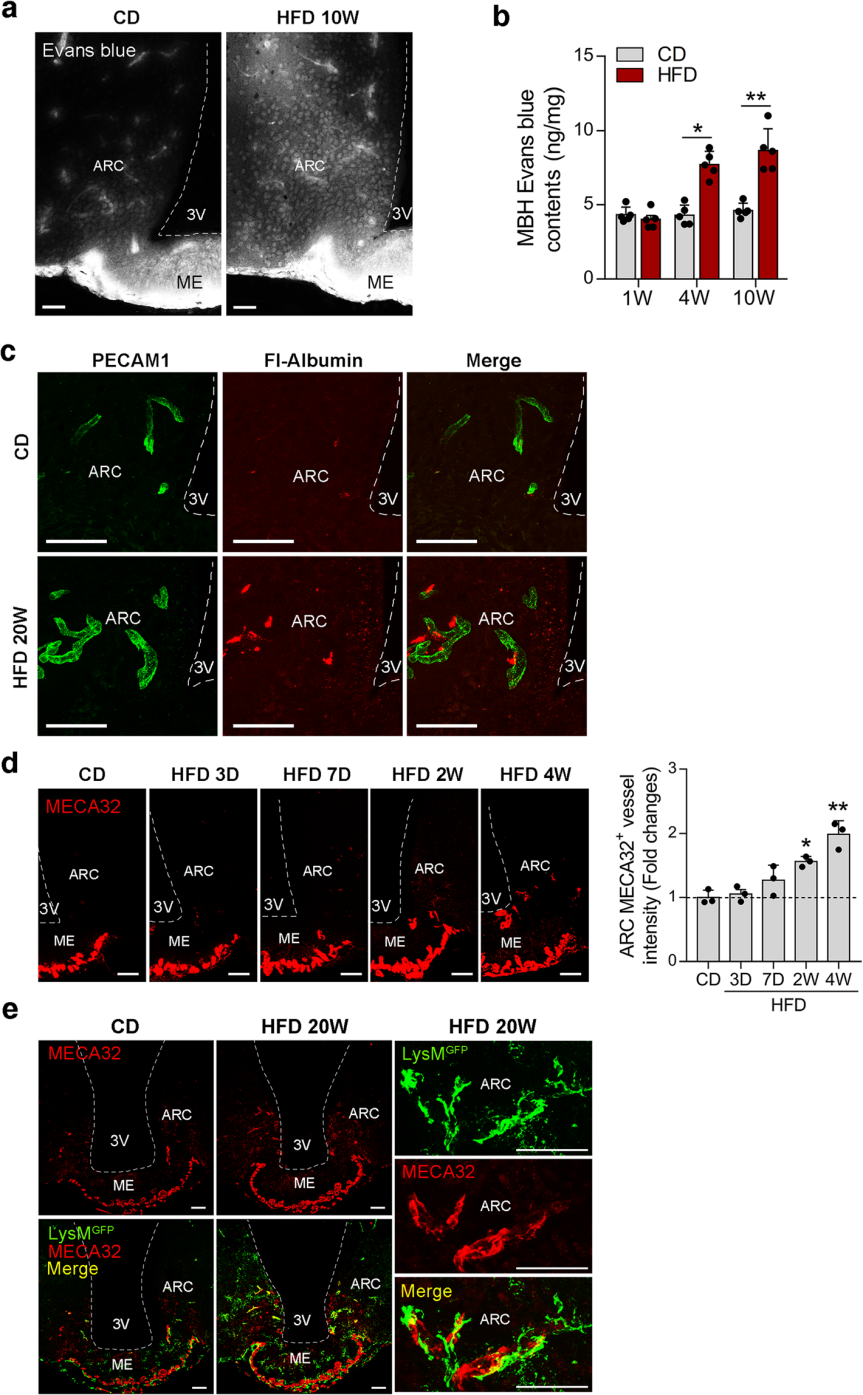
Increased vascular permeability in the ARC of HFD-induced obese mice. **a**, **b** Images and quantification analysis of Evans blue leakage in the hypothalamus of mice fed on a CD or HFD for the indicated periods. **c** Double fluorescence images of the vascular marker PECAM1 and fluorescence (Fl)-labeled albumin in the ARC of CD- and HFD-fed mice that received an intra-tail vein injection of Fl-albumin. **d** Representative images and quantification analysis of fenestrated vessel marker MECA32 expression in the hypothalamic ARC during the course of HFD feeding. **e** Double immunostaining of MECA32 and GFP showing activated LysM^GFP^ perivascular macrophages around MECA32^+^ permeable ARC blood vessels in 20-week HFD-fed mice. Scale bars, 50 μm. 3V, third ventricle; ARC, hypothalamic arcuate nucleus; MBH, mediobasal hypothalamus; ME, median eminence. Results are presented as a mean ± SEM. *p < 0.05 and **p < 0.01 vs. CD or between indicated groups

To test the vascular permeability of the hypothalamic ARC in DIO mice, we first injected a vessel-impermeable dye, Evans blue, into the tail veins of lean and obese mice fed on a CD or an HFD for the indicated periods. In the lean mice, Evans blue fluorescence was observed inside the hypothalamic blood vessels, although it leaked into the parenchyma in the circumventricular organ ME (Fig. 1a). In contrast, increased Evans blue leakage was observed in the ARC of 10-week HFD-fed mice. A time-course quantification study also demonstrated an increased Evans blue content in the MBH of obese mice on an HFD for 4 and 10 weeks (Fig. 1b). Consistently, increased extravasation of fluorescence-conjugated albumin was observed in the ARC of obese 20-week HFD-fed mice whereas no leakage was found in the lean controls (Fig. 1c). These data indicated that the BBB may become more permeable in the ARC of DIO mice. However, it is also possible that fluorescence-labeled albumin may infiltrate from permeable capillaries in the ME, which is in close proximity to the ARC.

We also stained hypothalamic slices with the fenestrated vessel marker MECA32 [10]. CD-fed lean mice showed a restricted distribution of MECA32-expressing permeable vessels in the external zone of the ME and no MECA32 expression in the ARC (Fig. 1d). However, ARC MECA32 expression significantly increased after 2 weeks of HFD feeding (Fig. 1d), indicating that the ARC microvasculature becomes permeable upon persistent HFD consumption.

In the hypothalamus, macrophages are mostly located in the perivascular space under normal conditions [14]. ARC perivascular macrophages (PVM) adopt a tubular shape without cellular processes [14]. However, upon activation by chronic exposure to HFD, they undergo morphological changes that include an enlarged body and branched processes [14]. MECA32 and GFP double immunostaining revealed activated LysM^GFP^ macrophages around the MECA32^+^ ARC vessels in 20-week HFD-fed LysM^GFP^ mice (Fig. 1e). Taken together, these data demonstrated increased vascular permeability and activated PVMs around permeable vessels in the ARC of DIO mice.

### No hypothalamic infiltration of circulating monocytes in DIO mice

**Fig. 2:**
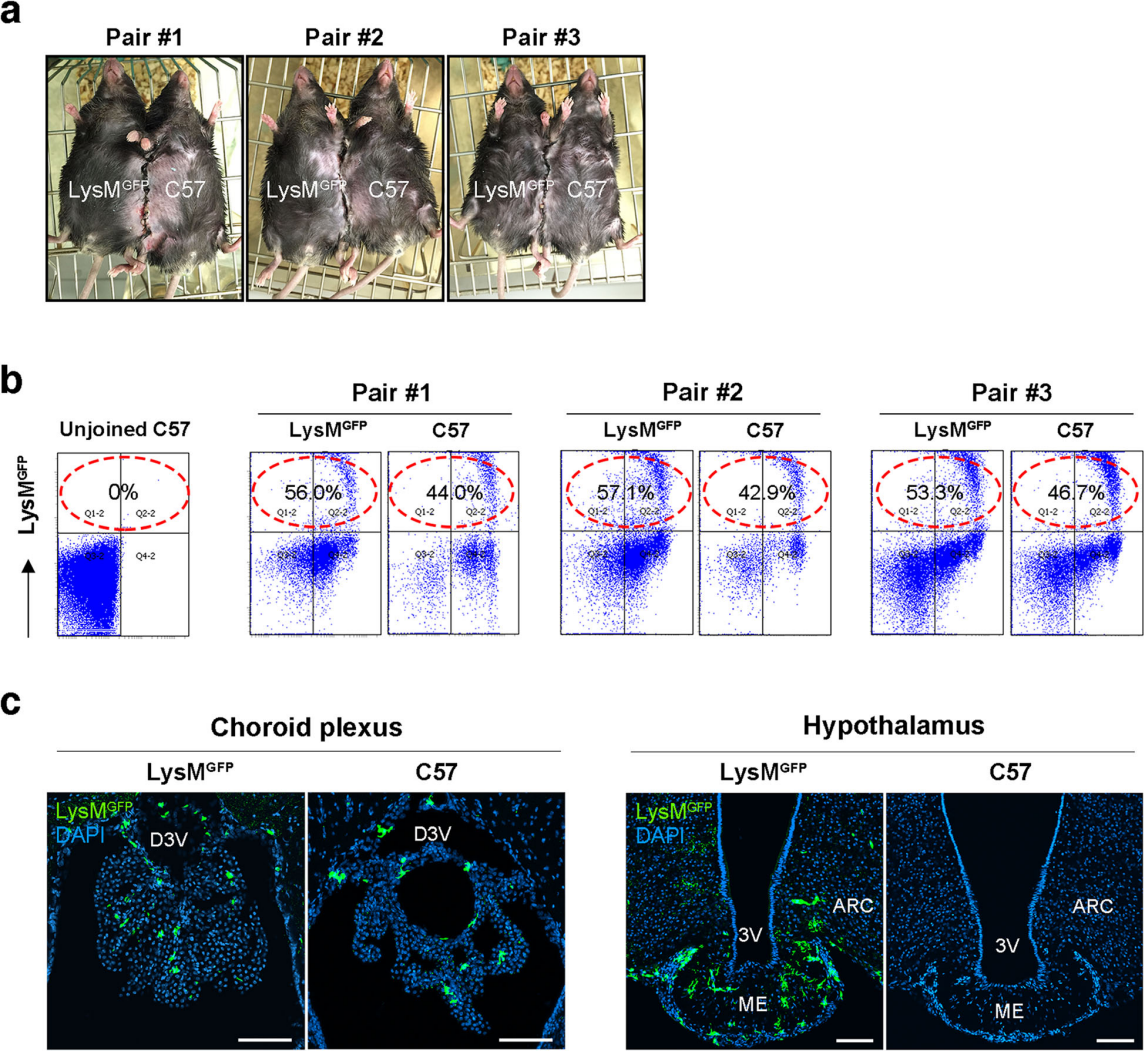
Parabiosis experiment indicating no recruitment of circulating LysM^GFP^ myeloid cells to the hypothalamus during 12-week HFD feeding. **a**, **b** Representative images of parabiosis between LysM^GFP^ and C57 mice and FACS analysis data confirming successful parabiosis. The percentages indicate the proportion of LysM^GFP^ cells among the peripheral blood monocytes. **c** Confocal images of GFP and DAPI immunostaining in the choroid plexus and hypothalamus of LysM^GFP^ and C57 parabionts showing that circulating LysM^GFP^ cells had infiltrated the choroid plexus but not the hypothalamus of the C57 mice. Scale bars, 100 μm. ARC, hypothalamic arcuate nucleus; D3V, dorsal third ventricle; ME, median eminence; 3V, third ventricle

To directly demonstrate enhanced recruitment of circulating monocytes to the ARC via permeable vessels, we performed a parabiosis experiment in which the circulation of LysM^GFP^ mice was connected to that of C57 mice by conjoining the frank skin (Fig. 2a). LysM^GFP^ partner-driven LysM^GFP^ cells were detected by FACS analysis in the blood of C57 mice at 6 weeks post-surgery (Fig. 2b), confirming that the parabiosis had been successful. Following a 12-week HFD, the parabionts were sacrificed and their brains were collected to determine whether LysM^GFP^ cells had infiltrated the hypothalamus of the C57 mice. In LysM^GFP^ mice, GFP-expressing cells were frequently found in the choroid plexus and in the hypothalamic ARC and ME (Fig. 2c). Of note, LysM^GFP^ cells were also detected in the choroid plexus of C57 parabionts fed on an HFD for 12 weeks (Fig. 2c). However, we could not detect any LysM^GFP^ cells in the hypothalamus of the C57 mice. These results suggested that LysM^GFP^ monocytes infiltrate the choroid plexus but may not enter the hypothalamus of DIO mice despite its vascular hyperpermeability.

**Fig. 3:**
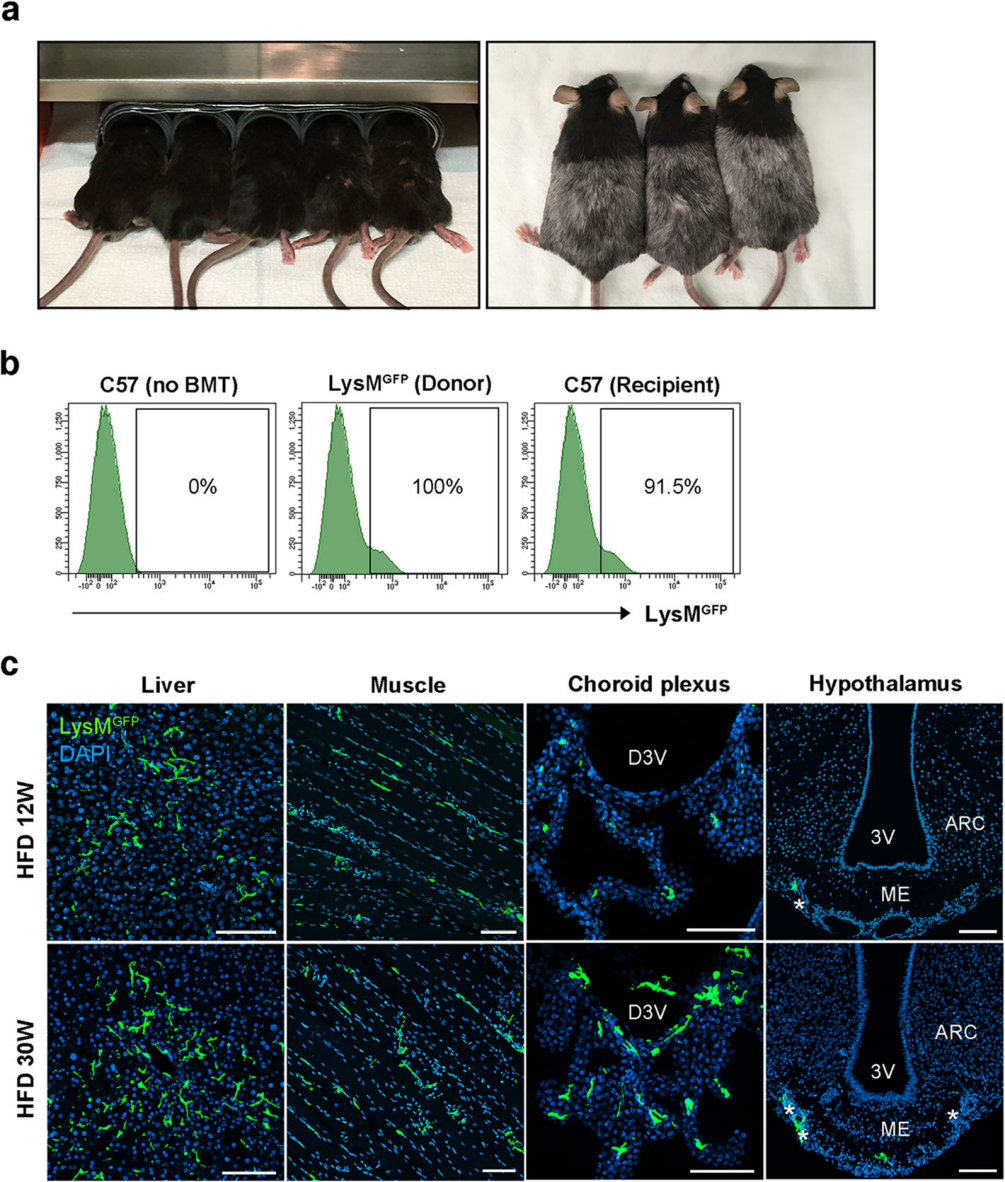
Bone marrow transplant study using LysM^GFP^ donor and C57 recipient mice. **a** Images of whole mouse body irradiation with a head shield prior to BMT. **b** Representative FACS data confirming a successful BMT. The percentages indicate the proportion of LysM^GFP^ cells among the peripheral blood monocytes. **c** Representative confocal images of GFP and DAPI immunostaining in the liver, skeletal muscle, choroid plexus, and hypothalamus of C57 recipients on a HFD for 12 and 30 weeks prior to sacrifice. Asterisks denote LysM^GFP^ cells along the meningeal lining. Scale bars, 100 μm. ARC, hypothalamic arcuate nucleus; D3V, dorsal third ventricle; ME, median eminence; 3V, third ventricle

We next conducted a BMT experiment to validate our parabiosis results. We established a BM chimera model by transplanting the BM from LysM^GFP^ mice to C57 mice. One week prior to this BMT, C57 mice received whole body irradiation with head shielding to prevent an artificial BBB breakdown by X-ray irradiation (Fig. 3a). BMT success was confirmed by the presence of LysM^GFP^ cells in the blood of BMT recipients at 6 weeks post-operation (Fig. 3b). Mice were fed with a HFD for either 12 weeks or 30 weeks before being sacrificed to observe LysM^GFP^ cells in the brain, liver, and skeletal muscle of the recipients. LysM^GFP^ cells were frequently found in the liver, muscle, and choroid plexus of the recipients that were fed with a HFD for 12 weeks or 30 weeks (Fig. 3c). Under both HFD feeding conditions, a few LysM^GFP^ cells were also found along the meningeal lining covering the ME (Fig. 3c). However, no LysM^GFP^ cells could be detected inside the hypothalamus of these animals, including the ARC. These findings strongly supported the notion that circulating monocytes do not significantly contribute to hypothalamic macrophage pool expansion under chronic HFD conditions.

### Hypothalamic recruitment of LysM^GFP^ cells during development

**Fig. 4:**
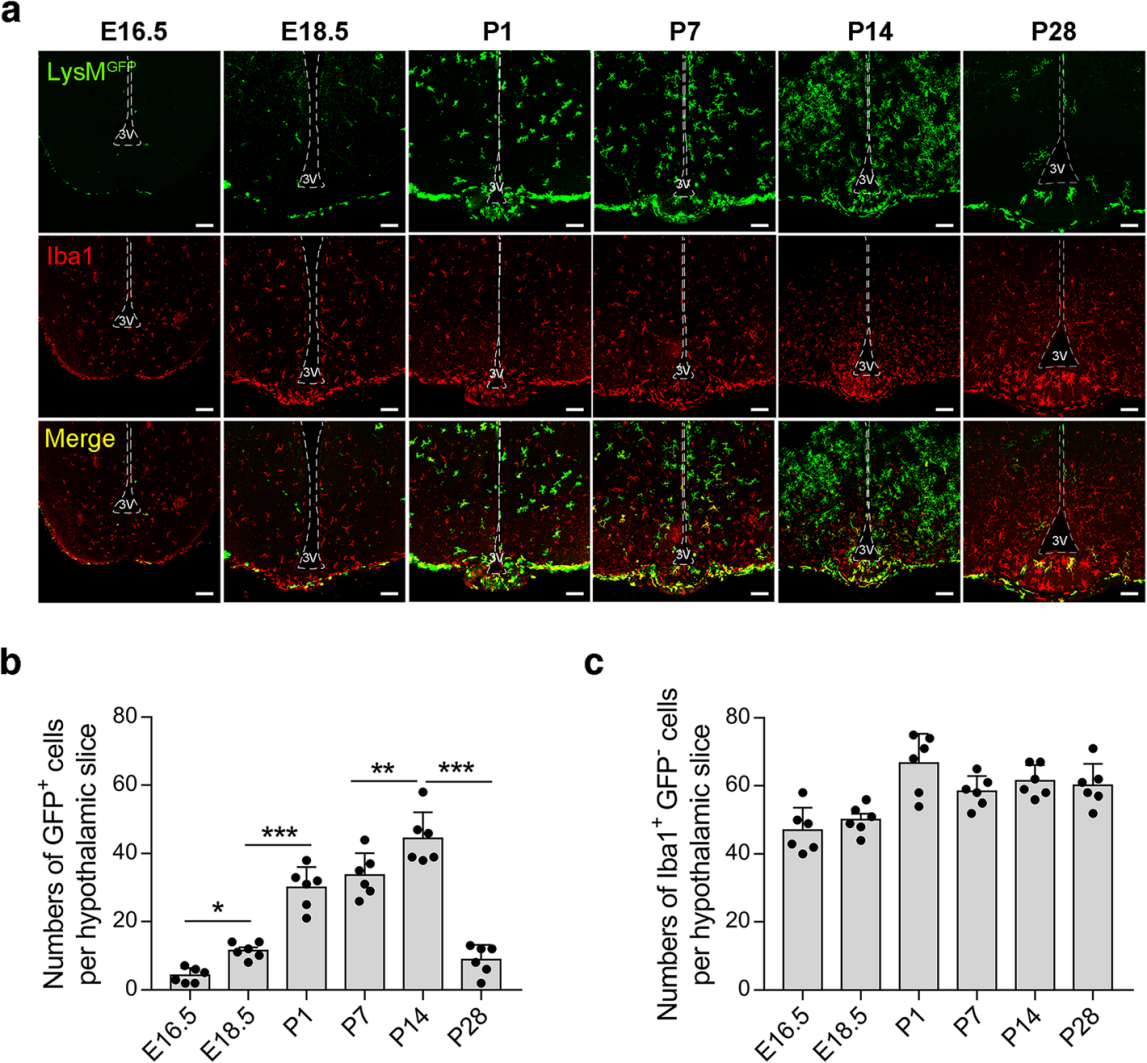
Infiltration of the hypothalamus by LysM^GFP^ cells during development. **a** Representative images of GFP and Iba1 double immunostaining in the brains of LysM^GFP^ embryos and neonates at embryonic days (E) 16.5 and 18.5 and postnatal days (P) 1, 7, 14, and 28. **b**, **c** Quantification of LysM^GFP^ cells and Iba1 alone-expressing microglia in the hypothalamus of LysM^GFP^ embryos and neonates. Scale bars, 100 μm. 3V, third ventricle. Results are presented as a mean ± SEM. *p < 0.05, **p < 0.01, and ***p < 0.001 between the indicated groups

Given the observed lack of any definite recruitment of LysM^GFP^ circulating monocytes to the hypothalamus, we next investigated whether these cells populate the hypothalamus during the developmental period. The brains of LysM^GFP^ mice were collected on embryonic days (E) 16.5 and 18.5 and on postnatal days (P) 1, 7, 14, and 28 for double immunostaining of GFP and the microglial marker Iba1. At E16.5, LysM^GFP^ cells were barely detectable in the hypothalamic area, although a small number could be observed along the meningeal lining (Fig. 4a, b). In contrast, many Iba1-expressing microglia were evident in the hypothalamic area at E16.5, which were thought to be yolk sac derived (Fig. 4a, c). Of note, a hypothalamic pool of LysM^GFP^ cells started to increase from E18.5 and dramatically expanded immediately after birth (Fig. 4a, b). During the mid-lactation period (P14), a huge expansion of LysM^GFP^ cells was observed in the hypothalamus, with an activated microglia-like morphology (Fig. 4a). These results indicated that these cells may be highly activated even when nourished by CD-fed dams. At P28, the hypothalamic LysM^GFP^ pool was profoundly reduced and had adopted the adult pattern of distribution (Fig. 4a, b), as reported previously [14]. In sharp contrast to the dynamic changes in the hypothalamic LysM^GFP^ cellular pool, the Iba1-alone expressing microglia pool maintained relatively consistent numbers during early postnatal life (Fig. 4a, c). Interestingly, a significant proportion of the meningeal and ME LysM^GFP^ cells coexpressed Iba1, whereas most of the LysM^GFP^ cells inside the hypothalamus did not (Fig. 4a).

## Discussion

We show from our current analyses that circulating LysM^GFP^ myeloid cells are not actively recruited to the hypothalamic ARC, even under the conditions of chronic HFD feeding. In contrast, a significant infiltration of LysM^GFP^ cells was observed in peripheral organs such as the liver and skeletal muscle, and also within CNS sites such as the choroid plexus and meninges adjacent to the hypothalamus. Hence, the observed lack of infiltration of BM- or systemic circulation-derived LysM^GFP^ myeloid cells in the hypothalamus was not due to a failure of BMT or parabiosis. This lack of hypothalamic infiltration of circulating LysM^GFP^ cells in DIO mice was unexpected and surprising as the ARC vasculature becomes permeable from the early course of HFD feeding.

In contrast to our current findings, a previous study has reported a massive infiltration by peripheral immune cells of multiple brain regions including the hypothalamus in DIO mice [20]. In that prior report however, the mice underwent whole body irradiation including the head prior to BMT. This head irradiation could have led to a breakdown of the BBB and thus artificially allowed immune cell entry to the brain [21]. These previous data should therefore be interpreted with caution. Valdearcos et al. have also reported a significant infiltration of the ARC by BM-derived myeloid cells under 4-week HFD feeding conditions using BMT [15]. In that report, the authors transplanted the BM from ubiquitin-GFP mice to C57 mice that had been irradiated with a head shield. BM-derived myeloid cells were subsequently detected in the hypothalamus using GFP and pan-myeloid marker CD68 double immunostaining. It was shown from that experiment that about 30% of the cells among the ARC CD68^+^ myeloid population in mice on a 4-week HFD had GFP expression. The authors pointed out that these cells were periphery-derived myeloid cells.

The reasons for the discrepancy between the results of our current analysis and those of the aforementioned previous study [15] are unclear at present. One possibility is that the GFP^+^ myeloid cells seen in the hypothalamus may be short-lived myeloid cells such as neutrophils or monocytes. Thus, they may neither differentiate to hypothalamic resident macrophages nor significantly contribute to the hypothalamic macrophage pool. In support of this possibility, PVMs in the brain maintain a stable cellular pool without substantial exchange with blood cells for 46 weeks [22]. This slow turnover of PVMs may preclude the possibility that BM cell recruitment contributes to a marked macrophage accumulation in the ARC upon HFD feeding for several weeks [14].

The local proliferation of tissue macrophages has recently emerged as an important mechanism for maintaining a tissue macrophage pool [23, 24]. For instance, adipose tissue macrophages undergo local cell division and maintain their numbers via in situ proliferation under conditions of monocyte depletion [23]. Consistently, we have recently reported using LysM^GFP^ mice that ARC PVMs undergo rapid-onset in situ proliferation during HFD feeding [14]. Our time-course 5-bromouridine experiment in that prior study revealed increased in situ proliferation of PVMs from a 1-week exposure to an HFD. Furthermore, we observed Ki67^+^ proliferating PVMs abutting the ARC blood vessels of the obese mice [14]. These findings indicated that upon HFD feeding, hypothalamic macrophages expand mainly through self-renewal, which is similar to yolk sac-derived microglia [25].

Finally, in our current study, we attempted to determine the point at which the hypothalamic LysM^GFP^ macrophage pool is established during development. The LysM^GFP^ myeloid cells were found to mainly populate the hypothalamus during the postnatal lactation period, when hematopoiesis shifts from the liver to the BM [26]. These results thus suggest that most of the hypothalamic LysM^GFP^ cells arise from the BM. This contrasts starkly with the yolk sac-derived microglia which migrate to the neuroepithelium at around E9.5. Notably also, the LysM^GFP^ pools become profoundly expanded and by morphological examination seem to be activated at P14. These findings are suggestive of a potential active role of these cells in the hypothalamic development and neural circuit formation events that occur during this period [27].

## Conclusion

BM-derived macrophages mostly enter the hypothalamus during the early postnatal period prior to the closure of the BBB. These cells may maintain their pool via local proliferation, even under conditions of chronic HFD feeding. Hence, recruitment of circulating monocytes may have a minor role in hypothalamic macrophage accumulation in the process of HFD-induced hypothalamic inflammation.

## Data Availability

The datasets used and/or analyzed during the current study are available from the corresponding author on reasonable request.

## Abbreviations

ARC: Hypothalamic arcuate nucleus
BBB: Blood-brain barrier
BM: Bone marrow
BMT: Bone marrow transplant
CD: Chow diet
CNS: Central nervous system
DIO: Diet-induced obesity
GFP: Green fluorescence protein
HFD: High-fat diet
LysM: Lysozyme M
MBH: Mediobasal hypothalamus
ME: Median eminence
PECAM1: Platelet endothelial cell adhesion molecule-1
PVM: Perivascular macrophage
